# The Role of RNA-Binding Protein HuR in Lung Cancer by RNA Sequencing Analysis

**DOI:** 10.3389/fgene.2022.813268

**Published:** 2022-04-05

**Authors:** Xiong Ye, Qiang Fu, Hui Xiao

**Affiliations:** ^1^ School of Clinical Medicine, Shanghai University of Medicine & Health Sciences, Shanghai, China; ^2^ Department of Respiratory and Critical Care Medicine, Shanghai General Hospital, Shanghai Jiaotong University, Shanghai, China

**Keywords:** lung cancer, human antigen R, proliferation, RNA sequencing analysis, prognosis

## Abstract

**Background:** The overexpression of human antigen R (HuR) has been proven in various types of cancer and is associated with the poor survival lung cancer patients. HuR overexpression stabilizes the mRNA of tumor-promoting genes by binding with 3′-UTR AU-rich elements. However, the role of HuR in the proliferation of lung cancer is unclear.

**Methods:** HuR expression was assessed using immunohistochemistry of tumor tissue samples from ten patients with lung cancer and ten patients with benign lung disease. Gene, protein, mRNA, and lncRNA changes in A549 HuR knockdown (KD) cells were assessed by single-cell RNA sequencing analysis. Furthermore, cell proliferation, migration, and invasion were determined by Cell Counting Kit-8 (CCK-8) assays and Transwell assays with or without Matrigel. The cell cycle was assessed by propidium iodide staining. The protein level, mRNA level and half-life of PLK1 were detected by western blotting and RT-qPCR.

**Results:** In clinical patients, the expression of HuR was significantly higher in lung cancer patients than in patients with benign lung disease. RNA sequencing analysis of A549 HuR knockdown cells revealed that the main function of HuR was related to ribonucleoprotein complex biogenesis. HuR was found to regulate signaling pathways mainly related to the spliceosome, RNA transport and the cell cycle. HuR KD suppressed the proliferation, migration and invasion of A549 cells, indicating its promotive role in these processes.

**Conclusion:** These results demonstrate that HuR plays an important role in the progression of lung cancer.

## Background

Lung cancer is a systemic disease that causes more than one-fourth of all cancer-related deaths worldwide (American Cancer Society Cancer Statistics, 2021). Thus, the identification of novel therapeutic molecular targets to inhibit lung cancer proliferation is urgently needed to improve patient survival.

The human antigen R (HuR), an ELAV protein contains 3′ RNA-binding domains and RNA stabilization by the AU-rich elements (AREs) (Papatheofani et al., 2021). This makes HuR an important RNA-binding protein that exerts pleiotropic effects on tumorigenesis and tumor development (Brody and Dixon, 2018; Dhir et al., 2019). HuR can alter the cellular function to proliferation (Yu et al., 2020), heat or hypoxia stress (Reglero et al., 2020; Deka and Saha, 2021), apoptotic pathway (Chen et al., 2020), immune cell differentiation (Yu et al., 2021), senescence (Liebig et al., 2020), pro-inflammatory gene (Ke et al., 2021), and the immune regulation (Liu et al., 2021a; Goutas et al., 2021) by its posttranscriptional influence on specific target mRNAs. HuR overexpression in lung cancer may regulate progression by controlling mRNA stability (Dixon et al., 2001; Denkert et al., 2006; Lim et al., 2009). However, the role of HuR in the proliferation of lung cancer is still unclear. Polo-like kinase 1 (PLK1) plays an important role in the initiation, maintenance, and completion of mitosis. Dysfunction of PLK1 may promote cancerous transformation and drive cancer progression (Liu et al., 2017; Montaudon et al., 2020; Shin et al., 2020).

Therefore, in this study, we assessed the expression of HuR in primary lung cancer patients and benign lung disease patients. In addition, we used a human lung adenocarcinoma cancer cell line (A549) to knockdown (KD) and overexpress HuR to determine whether HuR can influence the function of those cells by single-cell RNA sequencing analysis and *in vitro* experiments.

## Materials and Methods

### Patients and Clinical Samples

The present study was approved by the Ethics Review Board of Shanghai General Hospital affiliated with Shanghai Jiaotong University (Shanghai, China). Written informed consent was obtained from all patients before enrollment in the study. All records were anonymized to protect individual confidentiality. The tumor tissues were retrieved from paraffin-embedded blocks. The clinical-stage was determined according to the recommendations of the eighth International Association for the Study of Lung Cancer (Ettinger et al., 2021).

### Immunohistochemical Staining and Immunoreactivity Scoring

Immunohistochemical staining and immunoreactivity scoring procedures were performed as described previously (Xiao et al., 2022). In brief, Resected specimens (lung cancer tissue and benign lung disease tissue) were fixed in formalin and embedded in paraffin. The primary antibody, HuR, was incubated overnight, and the secondary antibody was for 30 min. The expression of HuR in cancer cells was evaluated by immunoreactivity scoring based on the intensity and extent of staining.

### Cell Lines and Culture Conditions

The human lung adenocarcinoma cell line (A549) was purchased from the Type Culture Collection of the Chinese Academy of Sciences, Shanghai, China. It was cultured in Ham’s F-12K medium (Gibco) with 10% fetal bovine serum (Gibco) and maintained at 37°C in a humidified incubator with 5% CO_2_.

### Cell Transfection

Cells (3×10^5^ cells/well) were plated in a 12-well plate with a growth medium and without antibiotics 1 day before transfection. Twenty pmol siRNA was diluted in 50 µL Opti-MEM™ Reduced Serum Medium (Gibco). Next, we diluted 1.5 µL Lipofectamine 2000 in 50 µL Opti-MEM Reduced Serum Medium. After 5 min of incubation at room temperature, the diluted siRNA was combined with the diluted Lipofectamine 2000 and incubated for 15 min at room temperature. The cultured cells were added to the siRNA-liposome complexes. The cells were collected 72 h later, and the gene expression of HuR was detected by real-time PCR to verify the interference effect. We chose three different siRNA sequences to find the most effective one; the primer sequences used were as follows: HuR siRNA 610 (forward 5′- CGU​UUA​UCC​GGU​UUG​ACA​ATT -3′, reverse 5′- UUG​UCA​AAC​CGG​AUA​AAC​GTT -3′), siRNA 1024 (forward 5′- GCU​UUG​UGA​CCA​UGA​CAA​ATT -3′, reverse 5′- UUU​GUC​AUG​GUC​ACA​AAG​CTT -3′), siRNA 1239 (forward 5′- CAA​GUG​UUU​GUC​UUU​GUC​UTT -3′, reverse 5′- AGA​CAA​AGA​CAA​ACA​CUU​GTT -3′).

### RNA Extraction and RT-PCR

Total RNA was extracted by using the TRIzol method. First-strand cDNA was synthesized using the PrimeScriptTM RT Reagent Kit (TAKARA). The primer sequences used were as follows: HuR (forward 5′-TAA​TCG​CCA​TAG​CCT​TCC​TAA-3′, reverse 5′-GGC​GTC​TGC​AAA​TGG​TTG​TA-3′), PLK1 (forward 5′-TGT​GCT​GTC​GGT​ATG​GAG​AG-3′, reverse 5′-TCT​GGT​ACA​CAG​GAC​TTG​CG-3′) and GAPDH (forward 5′-CAA TGA CCC CTT CAT TGA CC-3′, reverse 5′-GAC AAG CTT CCC GTT CTC AG-3′). RT-PCRs were performed using SYBR^®^ Premix Ex Taq™ II (TAKARA). Three independent experiments were performed, and the data are shown as the mean ± SEM.

### Western Blotting

SDS-PAGE was performed, and antibodies specific for HuR/ELAVL1 (1:1,000, Abcam), PLK1 (1:1,000, Abcam), and β-actin (1:10,000, Proteintech) were applied for western blotting. Goat anti-mouse IgG (H + L) and goat anti-rabbit IgG (H + L) with HRP were used as the secondary antibodies (1:1,000 Beyotime). The results were visualized using an enhanced chemiluminescence detection system (Biosharp). β-Actin functioned as the reference protein.

### Cell Proliferation Assay

Then, 100 μL of cell suspension of the A549 normal group and siRNA interfering group (5,000 cells/well) was seeded in a 96-well plate. The cells were preincubated for 24 h in a humidified incubator (37°C, 5% CO_2_) and incubated for an appropriate length of time (0, 24, 48, 72 h) in the incubator. Then, 10 μL of CCK-8 solution was added to each well (Beyotime, Shanghai, China). The plate was incubated for 4 h in an incubator, and the absorbance was measured at 450 nm with a microplate reader.

### Cell Migration and Invasion Detection

The Transwell assay was performed with the Transwell system of Corning Co., Ltd. Inserts containing filters were uncoated for the migration assays or coated with Matrigel for the invasion assays. Then, 500 µL complete medium with 10% serum was added to the lower chamber, and a total of 1 × 10^5^ cells were added to 200 μL serum-free medium in the upper chamber. After 24 h of incubation at 37°C, the cells that had migrated through the filters were fixed with 800 µL of 4% methanol for 10 min, stained with 800 µL of crystal violet for 15–30 min, and counted under a microscope.

### Cell Cycle

A549 cells and A549 HuR KD cells incubated with F-12K medium without FBS were seeded in 6-well plates overnight. The F-12K medium was changed to 10% FBS, and the cells were incubated for 24 h. The cells were harvested, and cellular DNA was stained with propidium iodide (PI) solution. Fluorescence was measured with a flow cytometer (FACS Aria II, BD Bioscience, United States). Different cell cycle phases were distinguished based on the DNA content. DNA histograms were analyzed by ModFit 5.0 software.

### RNA Sequencing Analysis

RNA degradation and contamination were monitored on 1% agarose gels. RNA purity was checked using a NanoPhotometer^®^ spectrophotometer.

(IMPLEN, CA, United States). RNA integrity was assessed using the RNA Nano 6000 Assay Kit of the Bioanalyzer 2,100 system (Agilent Technologies, CA, United States). A total amount of 1 μg RNA per sample was used as input material for the RNA sample preparations. Sequencing libraries were generated using the NEBNext^®^ UltraTM RNA Library Prep Kit for Illumina^®^ (NEB, United States) following the manufacturer’s recommendations, and index codes were added to attribute sequences to each sample.

### Statistical Analysis

All values are expressed as the mean ± SEM for each experiment or a representative cell culture-based experiment. All statistical analyses were performed using GraphPad Prism 6.0. The student’s *t*-test was used to analyze the difference between the two groups. The Pearson’s test was used to analyze the correlation between the two samples. For all tests, the significance threshold was set at *p* ≤ 0.05.

## Results

### HuR is Overexpressed in Primary Lung Cancer Patients and Associated With a Poor Prognosis

**FIGURE 1:**
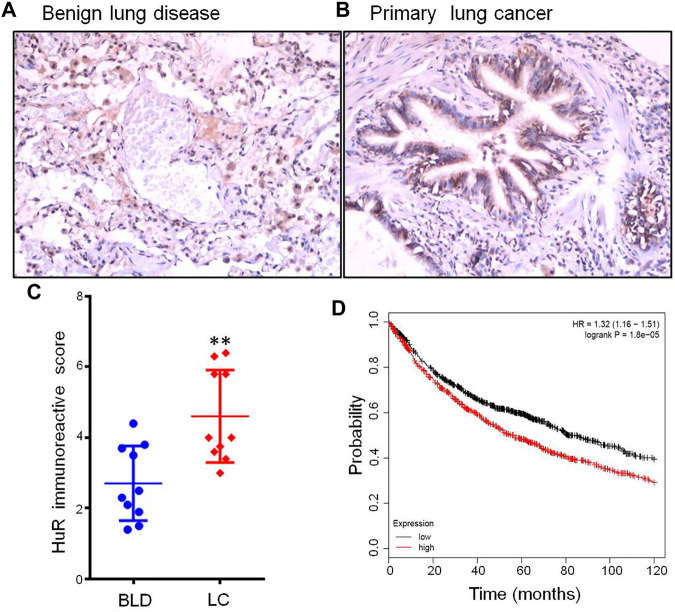
HuR expression in lung cancer patients and benign lung disease patients was detected using immunohistochemistry. HuR was stained in samples from patients with **(A)** benign lung disease (200 × magnification) and **(B)** lung cancer (200 × magnification) by IHC. **(C)** The IRS of HuR in benign lung disease and lung cancer samples. **(D)** Kaplan-Meier curve of overall survival according to HuR expression in the TCGA. There were 962 lung cancer patients with high expression among a total of 1926 patients, and lung cancer patients with high expression of HuR had a poor prognosis (*p* = 1.8e-05). **p < 0.01. BLD, benign lung disease; LC, lung cancer.

The immunohistochemical expression of HuR and IRS is shown in Figure 1, comparing representative samples of benign lung disease (Figure 1A) and primary lung cancer (Figure 1B). In the lung cancer slide, several cells that were positive for HuR were observed. Furthermore, Figure 1C shows the IRS of HuR expression in patients with lung cancer using IHC compared with benign lung disease (*p <* 0.01). Therefore, the overexpression of HuR is correlated with cancer. Analysis of The Cancer Genome Atlas (TCGA) database and Kaplan-Meier analysis of overall survival of 962 lung cancer patients with high HuR expression compared with 964 lung cancer patients with low HuR expression revealed that high expression of HuR indicated a poor prognosis (*p* = 1.8e-05) (Figure 1D).

### The Main Function of HuR in A549 Cells According to Single-Cell RNA Sequencing Analysis

**FIGURE 2:**
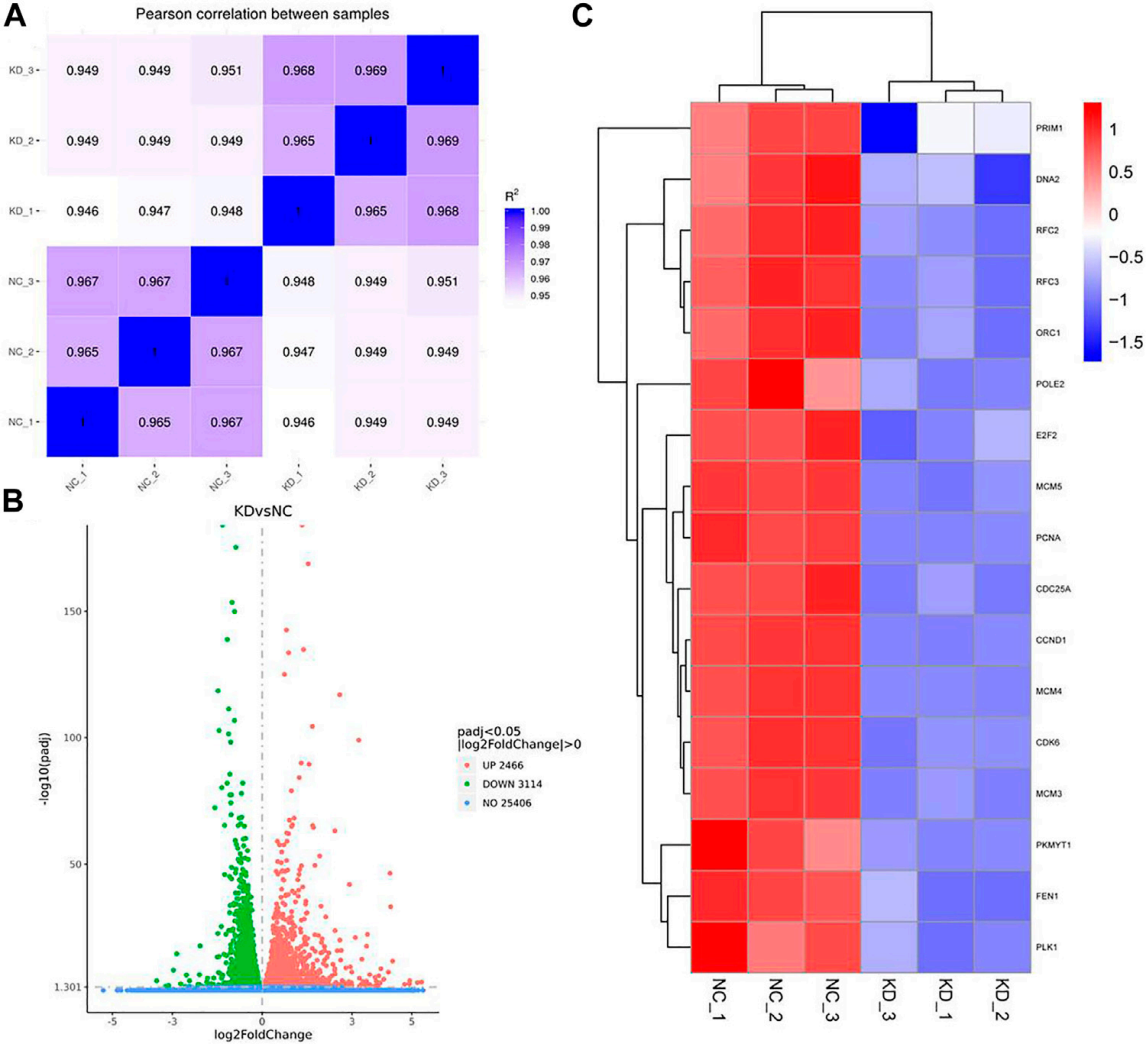
Heatmap showing the expression of genes in the lung cancer cell line A549 according to single-cell RNA sequencing. Cells are grouped by HuR KD status. **(A)**. Pearson correlation between HuR KD A549 cells and the control group. **(B)**. HuR KD A549 cells were analyzed by RNA sequencing, which revealed 2,466 upregulated genes and 3,114 downregulated genes. **(C)** The gene heatmap shows the expression of major genes involved in the cell cycle between the two groups.

**FIGURE 3:**
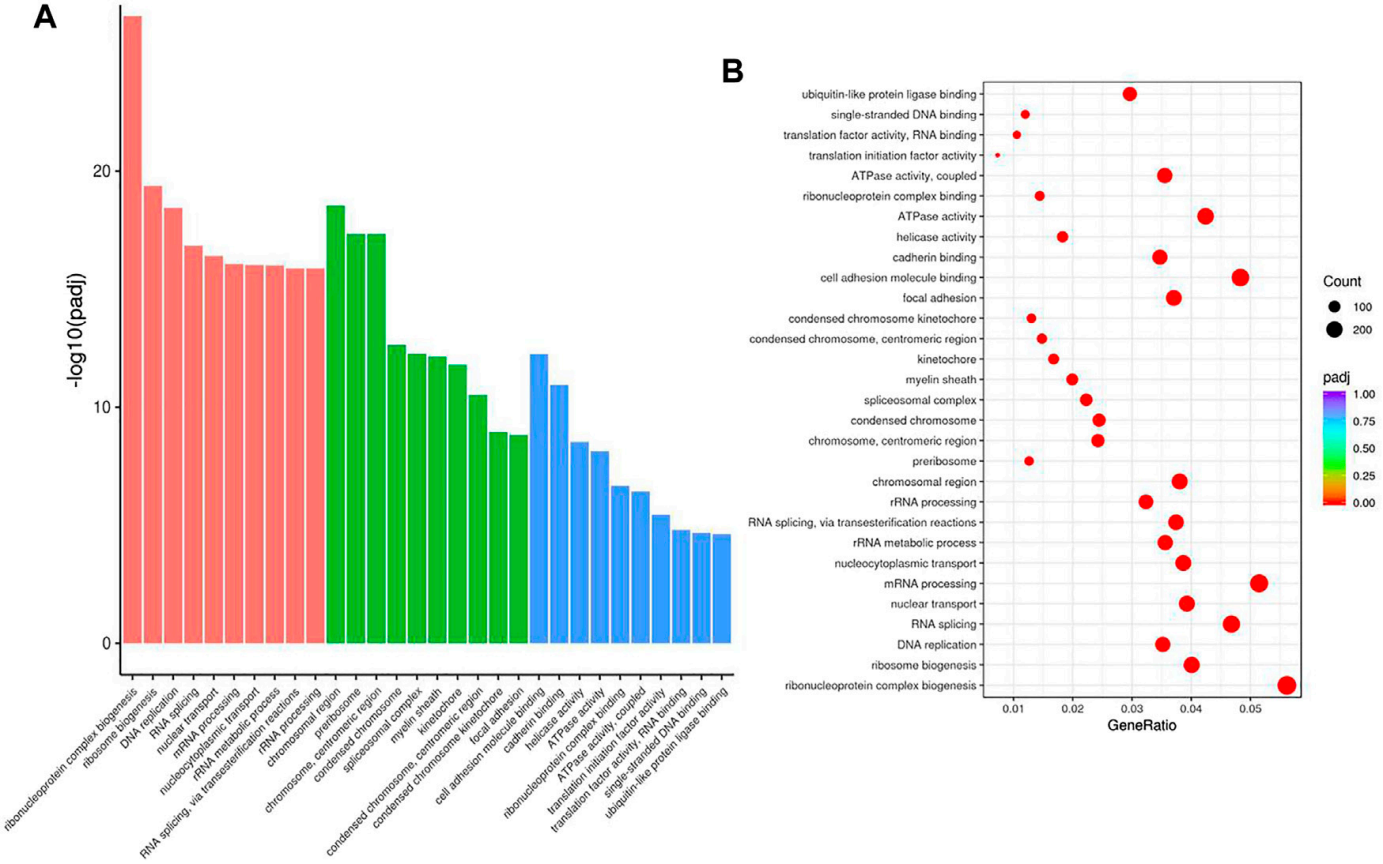
The cell functions enriched for significant differentially expressed genes between the HuR KD A549 cells and control cells. The histogram of GO analysis **(A)** combined with the bubble chart **(B)** shows that HuR mainly regulates the synthesis of ribonucleoprotein protein complexes.

**FIGURE 4:**
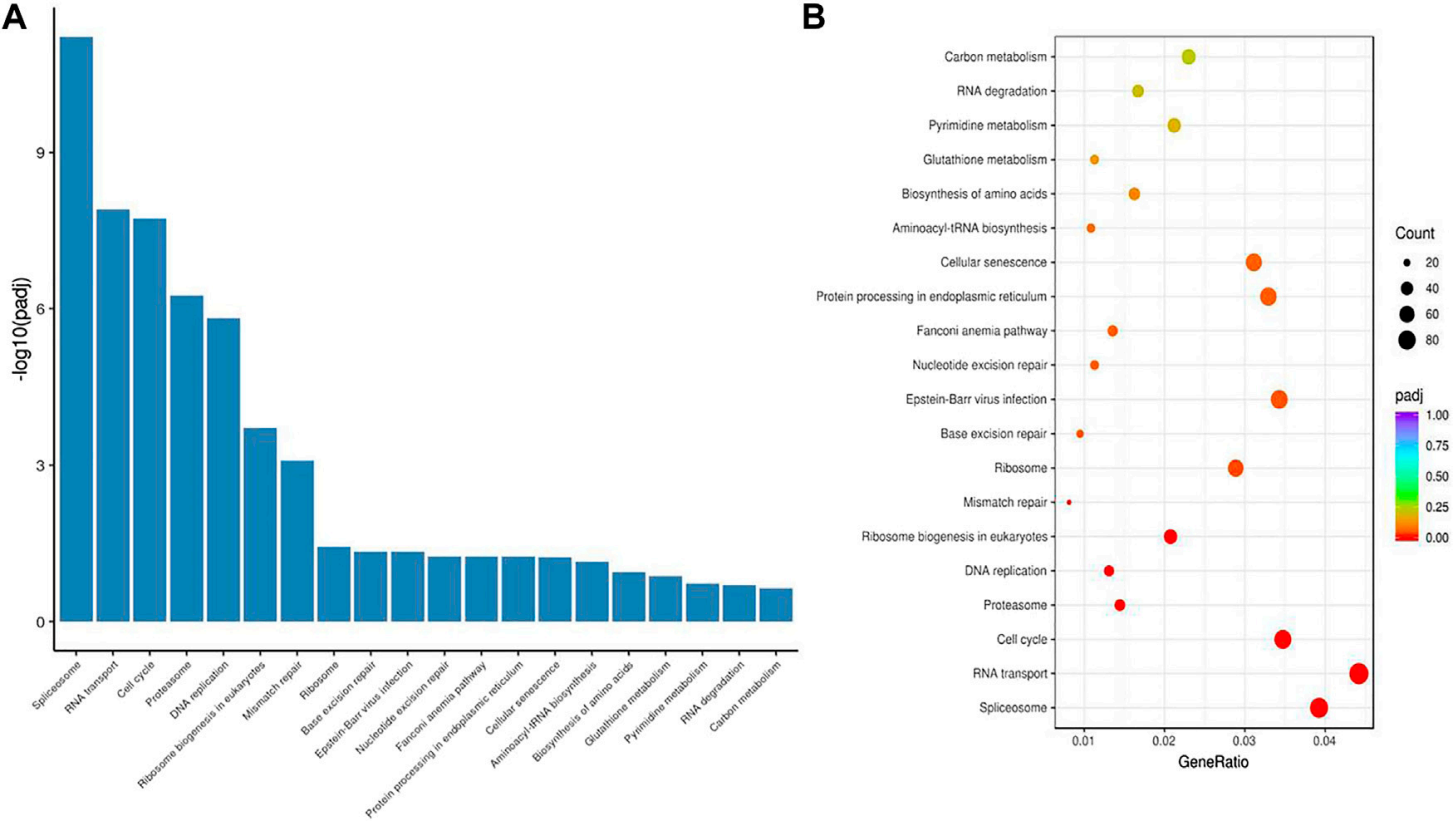
The signaling pathways enriched for significant differentially expressed genes in HuR KD A549 cells versus control cells. The histogram **(A)** and bubble chart **(B)** of the KEGG results show that HuR mainly regulates pathways related to the spliceosome, RNA transport, and the cell cycle.

To examine the function of HuR in lung adenocarcinoma cells, the lung adenocarcinoma cell line A549 was used, and we performed HuR KD for single-cell RNA sequencing analysis. First, the correlation between the two samples was checked by Pearson’s test. We found that 2,466 genes were upregulated and 3,114 genes were downregulated in HuR KD A549 cells versus control cells, as shown in the heatmap (Figure 2). Figure 3 indicates that the main functions of HuR in A549 cells were related to ribonucleoprotein complex biogenesis. The involved signaling pathways were included those related to spliceosomes, RNA transport, and the cell cycle (Figure 4). Table 1 and Table 2 show the changes in lncRNAs and microRNAs in A549 HuR KD cells.

**TABLE 1:** All microRNA changed in HuR Knockdown A549 cell by RNA Sequencing analysis.

Gene ID	Gene name	Gene length	Gene biotype	Gene description
ENSG00000283498	MIR1244-2	85	miRNA	microRNA 1,244-2 (Source:HGNC Symbol; Acc:HGNC:38321)
ENSG00000207652	MIR621	96	miRNA	microRNA 621 (Source:HGNC Symbol; Acc:HGNC:32877)
ENSG00000263597	MIR3936	110	miRNA	microRNA 3,936 (Source:HGNC Symbol; Acc:HGNC:38947)
ENSG00000265820	MIR3177	82	miRNA	microRNA 3,177 (Source:HGNC Symbol; Acc:HGNC:38364)
ENSG00000210140	MT-TC	66	Mt_tRNA	Mitochondrially encoded tRNA cysteine (Source:HGNC Symbol; Acc:HGNC:7477)
ENSG00000212232	SNORD17	237	snoRNA	Small nucleolar RNA, C/D box 17 (Source:HGNC Symbol; Acc:HGNC:32713)
ENSG00000238840	RF00096	133	snoRNA	
ENSG00000239148	RF00096	133	snoRNA	
ENSG00000201684	RN7SKP239	295	misc_RNA	RNA, 7SK small nuclear pseudogene 239 (Source:HGNC Symbol; Acc:HGNC:45963)
ENSG00000210135	MT-TN	73	Mt_tRNA	Mitochondrially encoded tRNA asparagine (Source:HGNC Symbol; Acc:HGNC:7493)
ENSG00000281344	HELLPAR	205,012	macro_lncRNA	HELLP associated long non-coding RNA (Source:HGNC Symbol; Acc:HGNC:43984)
ENSG00000210194	MT-TE	69	Mt_tRNA	Mitochondrially encoded tRNA glutamic acid (Source:HGNC Symbol; Acc:HGNC:7479)

**TABLE 2:** All lincRNA changed in HuR Knockdown A549 cell by RNA Sequencing analysis.

Gene ID	Gene name	Gene length	Gene biotype	Gene description
ENSG00000251562	MALAT1	8,829	lincRNA	Metastasis associated lung adenocarcinoma transcript 1 (Source:HGNC Symbol; Acc:HGNC:29665)
ENSG00000245532	NEAT1	22,767	lincRNA	Nuclear paraspeckle assembly transcript 1 (Source:HGNC Symbol; Acc:HGNC:30815)
ENSG00000214548	MEG3	23,224	lincRNA	Maternally expressed 3 (Source:HGNC Symbol; Acc:HGNC:14575)
ENSG00000268621	IGFL2-AS1	1751	lincRNA	IGFL2 antisense RNA 1 (Source:HGNC Symbol; Acc:HGNC:52559)
ENSG00000224259	LINC01133	3,155	lincRNA	Long intergenic non-protein coding RNA 1133 (Source:HGNC Symbol; Acc:HGNC:49447)
ENSG00000273038	AL365203.2	2057	lincRNA	Novel transcript
ENSG00000273760	AC245041.1	826	lincRNA	Novel transcript
ENSG00000250337	PURPL	2,638	lincRNA	p53 upregulated regulator of p53 levels (Source:HGNC Symbol; Acc:HGNC:48995)
ENSG00000273033	LINC02035	5,476	lincRNA	Long intergenic non-protein coding RNA 2035 (Source:HGNC Symbol; Acc:HGNC:52875)
ENSG00000260604	AL590004.3	7,060	lincRNA	Novel transcript
ENSG00000285517	LINC00941	18,914	lincRNA	Long intergenic non-protein coding RNA 941 (Source:NCBI gene; Acc:100287314)
ENSG00000272168	CASC15	13,289	lincRNA	Cancer susceptibility 15 (Source:HGNC Symbol; Acc:HGNC:28245)
ENSG00000281649	EBLN3P	6,483	lincRNA	Endogenous Bornavirus-like nucleoprotein 3, pseudogene (Source:HGNC Symbol; Acc:HGNC:50682)
ENSG00000163009	C2orf48	2,502	lincRNA	Chromosome 2 open reading frame 48 (Source:HGNC Symbol; Acc:HGNC:26322)
ENSG00000240875	LINC00886	3,822	lincRNA	Long intergenic non-protein coding RNA 886 (Source:HGNC Symbol; Acc:HGNC:48572)
ENSG00000272068	AL365181.2	3,222	lincRNA	Novel transcript
ENSG00000223749	MIR503HG	1,678	lincRNA	MIR503 host gene (Source:HGNC Symbol; Acc:HGNC:28258)
ENSG00000180422	LINC00304	3,961	lincRNA	Long intergenic non-protein coding RNA 304 (Source:HGNC Symbol; Acc:HGNC:26713)
ENSG00000261824	LINC00662	7,958	lincRNA	Long intergenic non-protein coding RNA 662 (Source:HGNC Symbol; Acc:HGNC:27122)
ENSG00000203721	LINC00862	3,174	lincRNA	Long intergenic non-protein coding RNA 862 (Source:HGNC Symbol; Acc:HGNC:21901)
ENSG00000247134	AC090204.1	765	lincRNA	Novel transcript
ENSG00000279501	CR392039.3	227	lincRNA	Novel transcript
ENSG00000235770	LINC00607	5,628	lincRNA	Long intergenic non-protein coding RNA 607 (Source:HGNC Symbol; Acc:HGNC:43944)
ENSG00000227640	SOX21-AS1	3,287	lincRNA	SOX21 antisense divergent transcript 1 (Source:HGNC Symbol; Acc:HGNC:39807)
ENSG00000197182	MIRLET7BHG	5,728	lincRNA	MIRLET7B host gene (Source:HGNC Symbol; Acc:HGNC:37189)
ENSG00000261786	AC006058.1	5,067	lincRNA	Novel transcript
ENSG00000251432	AC108062.1	3,283	lincRNA	Uncharacterized LOC100507487 (Source:NCBI gene; Acc:100507487)
ENSG00000276850	AC245041.2	2,467	lincRNA	Novel transcript
ENSG00000238266	LINC00707	9,833	lincRNA	Long intergenic non-protein coding RNA 707 (Source:HGNC Symbol; Acc:HGNC:44691)
ENSG00000187185	AC092118.1	2,114	lincRNA	Uncharacterized LOC388282 (Source:NCBI gene; Acc:388282)
ENSG00000249550	LINC01234	4,484	lincRNA	Long intergenic non-protein coding RNA 1234 (Source:HGNC Symbol; Acc:HGNC:49757)
ENSG00000229425	AJ009632.2	8,941	lincRNA	Uncharacterized LOC101927745 (Source:NCBI gene; Acc:101927745)
ENSG00000264112	AC015813.1	5,812	lincRNA	Novel transcript
ENSG00000260032	NORAD	5,339	lincRNA	Non-coding RNA activated by DNA damage (Source:HGNC Symbol; Acc:HGNC:44311)
ENSG00000230590	FTX	28,487	lincRNA	FTX transcript, XIST regulator (Source:HGNC Symbol; Acc:HGNC:37190)
ENSG00000267922	AC007785.1	332	lincRNA	Novel transcript
ENSG00000226137	BAIAP2-DT	4,773	lincRNA	BAIAP2 divergent transcript (Source:HGNC Symbol; Acc:HGNC:44342)
ENSG00000248508	SRP14-AS1	2,992	lincRNA	SRP14 antisense RNA1 (head to head) (Source:HGNC Symbol; Acc:HGNC:48619)
ENSG00000268362	AC092279.1	2,191	lincRNA	Novel transcript
ENSG00000262468	LINC01569	5,237	lincRNA	Long intergenic non-protein coding RNA 1569 (Source:HGNC Symbol; Acc:HGNC:51380)
ENSG00000180066	C10orf91	2040	lincRNA	Chromosome 10 open reading frame 91 (putative) (Source:HGNC Symbol; Acc:HGNC:27275)
ENSG00000253130	AC114550.1	1,075	lincRNA	Novel transcript
ENSG00000260260	SNHG19	342	lincRNA	Small nucleolar RNA host gene 19 (Source:HGNC Symbol; Acc:HGNC:49574)
ENSG00000232324	AC008440.3	555	lincRNA	Novel transcript
ENSG00000249464	LINC01091	7,779	lincRNA	Long intergenic non-protein coding RNA 1091 (Source:HGNC Symbol; Acc:HGNC:27721)
ENSG00000278730	AC005332.6	2,921	lincRNA	Novel transcript
ENSG00000205015	LINC02138	1,326	lincRNA	Long intergenic non-protein coding RNA 2138 (Source:HGNC Symbol; Acc:HGNC:52998)
ENSG00000232956	SNHG15	3,647	lincRNA	Small nucleolar RNA host gene 15 (Source:HGNC Symbol; Acc:HGNC:27797)
ENSG00000225783	MIAT	11,829	lincRNA	Myocardial infarction associated transcript (Source:HGNC Symbol; Acc:HGNC:33425)
ENSG00000275438	AL031008.1	541	lincRNA	Novel transcript
ENSG00000260804	LINC01963	3,148	lincRNA	Long intergenic non-protein coding RNA 1963 (Source:HGNC Symbol; Acc:HGNC:25283)
ENSG00000243701	DUBR	4,206	lincRNA	DPPA2 upstream binding RNA (Source:HGNC Symbol; Acc:HGNC:48569)
ENSG00000266835	GAPLINC	1,051	lincRNA	Gastric adenocarcinoma associated, positive CD44 regulator, long intergenic non-coding RNA (Source:HGNC Symbol; Acc:HGNC:51308)
ENSG00000262454	MIR193BHG	6,049	lincRNA	MIR193B host gene [Source:HGNC Symbol; Acc:HGNC:51945]
ENSG00000245571	FAM111A-DT	2,645	lincRNA	FAM111A divergent transcript (Source:HGNC Symbol; Acc:HGNC:53752)
ENSG00000272079	AC004233.3	1,269	lincRNA	Novel transcript
ENSG00000230454	U73166.1	2,625	lincRNA	Novel transcript
ENSG00000231131	LNCAROD	2007	lincRNA	lncRNA activating regulator of DKK1 (Source:HGNC Symbol; Acc:HGNC:50913)
ENSG00000232677	LINC00665	7,201	lincRNA	long intergenic non-protein coding RNA 665 (Source:HGNC Symbol; Acc:HGNC:44323)
ENSG00000275720	AC243830.2	630	lincRNA	Novel transcript
ENSG00000278765	AC004477.3	590	lincRNA	Novel transcript
ENSG00000197813	AC011450.1	615	lincRNA	Novel transcript
ENSG00000283073	SMUG1-AS1	1734	lincRNA	SMUG1 antisense RNA 1 (Source:HGNC Symbol; Acc:HGNC:53307)
ENSG00000237523	LINC00857	2,171	lincRNA	long intergenic non-protein coding RNA 857 (Source:HGNC Symbol; Acc:HGNC:45114)
ENSG00000282849	AL359834.1	638	lincRNA	Novel transcript
ENSG00000275759	AC026367.3	590	lincRNA	Novel transcript
ENSG00000276445	AC005393.1	584	lincRNA	Novel transcript
ENSG00000251365	AC122710.2	2,933	lincRNA	Novel transcript
ENSG00000236947	AL139412.1	990	lincRNA	Novel transcript
ENSG00000244649	LINC02086	3,858	lincRNA	Long intergenic non-protein coding RNA 2086 (Source:HGNC Symbol; Acc:HGNC:52936)
ENSG00000253227	AC090192.2	553	lincRNA	Novel transcript
ENSG00000249395	CASC9	1,473	lincRNA	Cancer susceptibility 9 (Source:HGNC Symbol; Acc:HGNC:48906)
ENSG00000225269	LINC00705	1745	lincRNA	Long intergenic non-protein coding RNA 705 (Source:HGNC Symbol; Acc:HGNC:27874)
ENSG00000234608	MAPKAPK5-AS1	2,290	lincRNA	MAPKAPK5 antisense RNA 1 (Source:HGNC Symbol; Acc:HGNC:24091)
ENSG00000267454	ZNF582-AS1	2037	lincRNA	ZNF582 antisense RNA 1 (head to head) (Source:HGNC Symbol; Acc:HGNC:25213)
ENSG00000276814	AC004801.6	978	lincRNA	Novel transcript
ENSG00000247095	MIR210HG	2,321	lincRNA	MIR210 host gene (Source:HGNC Symbol; Acc:HGNC:39524)
ENSG00000260388	LINC00562	2,470	lincRNA	Long intergenic non-protein coding RNA 562 (Source:HGNC Symbol; Acc:HGNC:43706)
ENSG00000261804	AC007342.4	1,106	lincRNA	Uncharacterized LOC102723373 (Source:NCBI gene; Acc:102723373)
ENSG00000234840	LINC01239	2,568	lincRNA	Long intergenic non-protein coding RNA 1239 (Source:HGNC Symbol; Acc:HGNC:49796)
ENSG00000182165	TP53TG1	1,023	lincRNA	TP53 target 1 (Source:HGNC Symbol; Acc:HGNC:17026)
ENSG00000272180	AC011306.1	1,275	lincRNA	Novel transcript

### HuR Promotes Cell Proliferation, Migration, and Invasion

**FIGURE 5:**
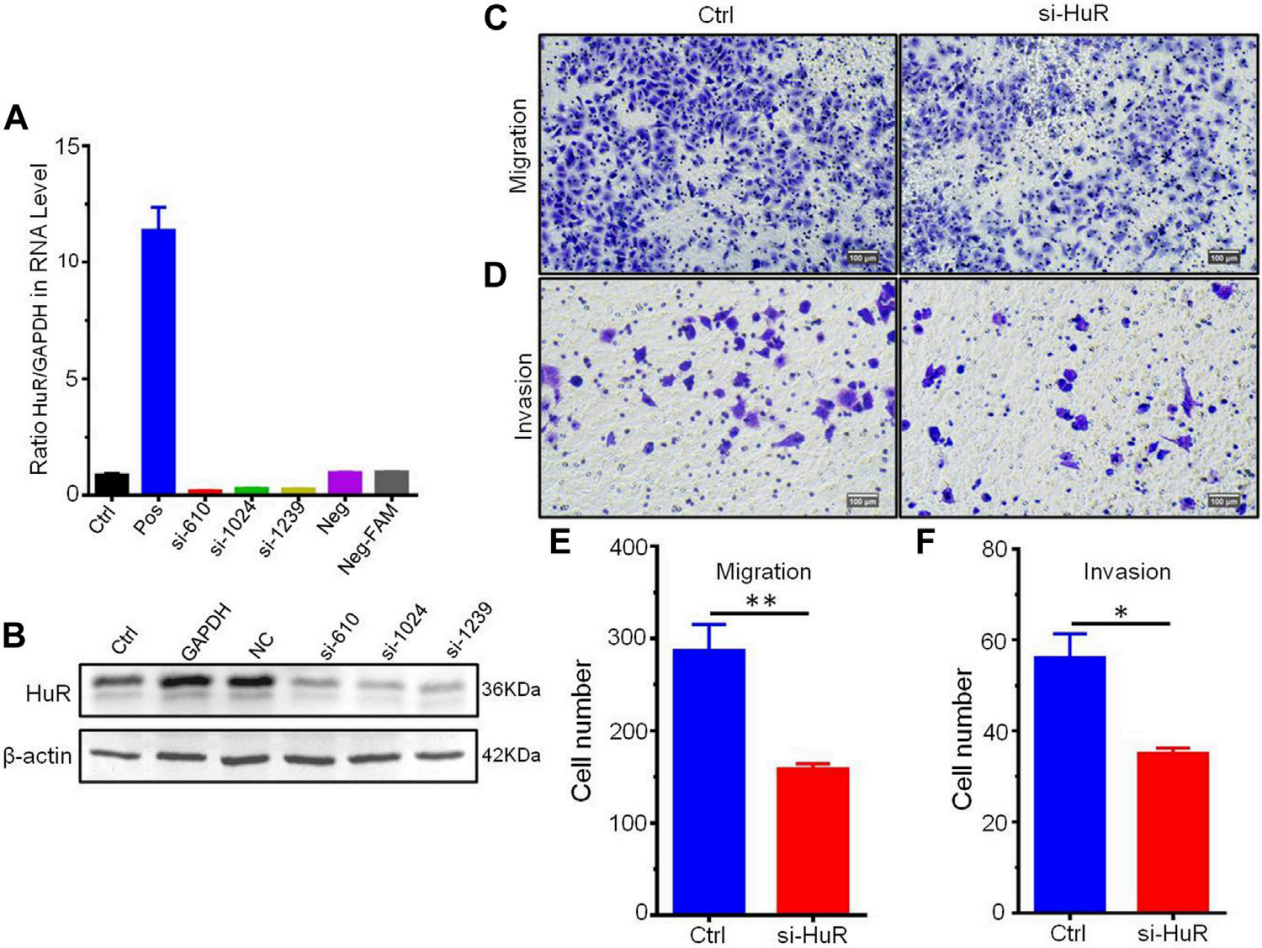
HuR promotes the migration and invasion of A549 cells. Migration assay of A549 HuR KD cells (1*10^5^/well) compared with A549 cells as a control. The expression of HuR genes was detected by western blotting and real-time PCR to verify the interference effect of siHuRs in A549 cells **(A,B)**. Cells that migrated into the lower chamber were photographed **(C)** and quantified **(E)**. Matrigel cell invasion assay of A549 HuR KD cells (1*10^5^/well) compared with A549 cells as a control for 24 h. Cells that invaded through the Matrigel were photographed **(D)** and quantified **(F)**. Scale bar 100 μm *p < 0.05, **p < 0.01, ***p < 0.001. Data are represented as the mean ± SEM. All experiments were repeated at least three times.

**FIGURE 6:**
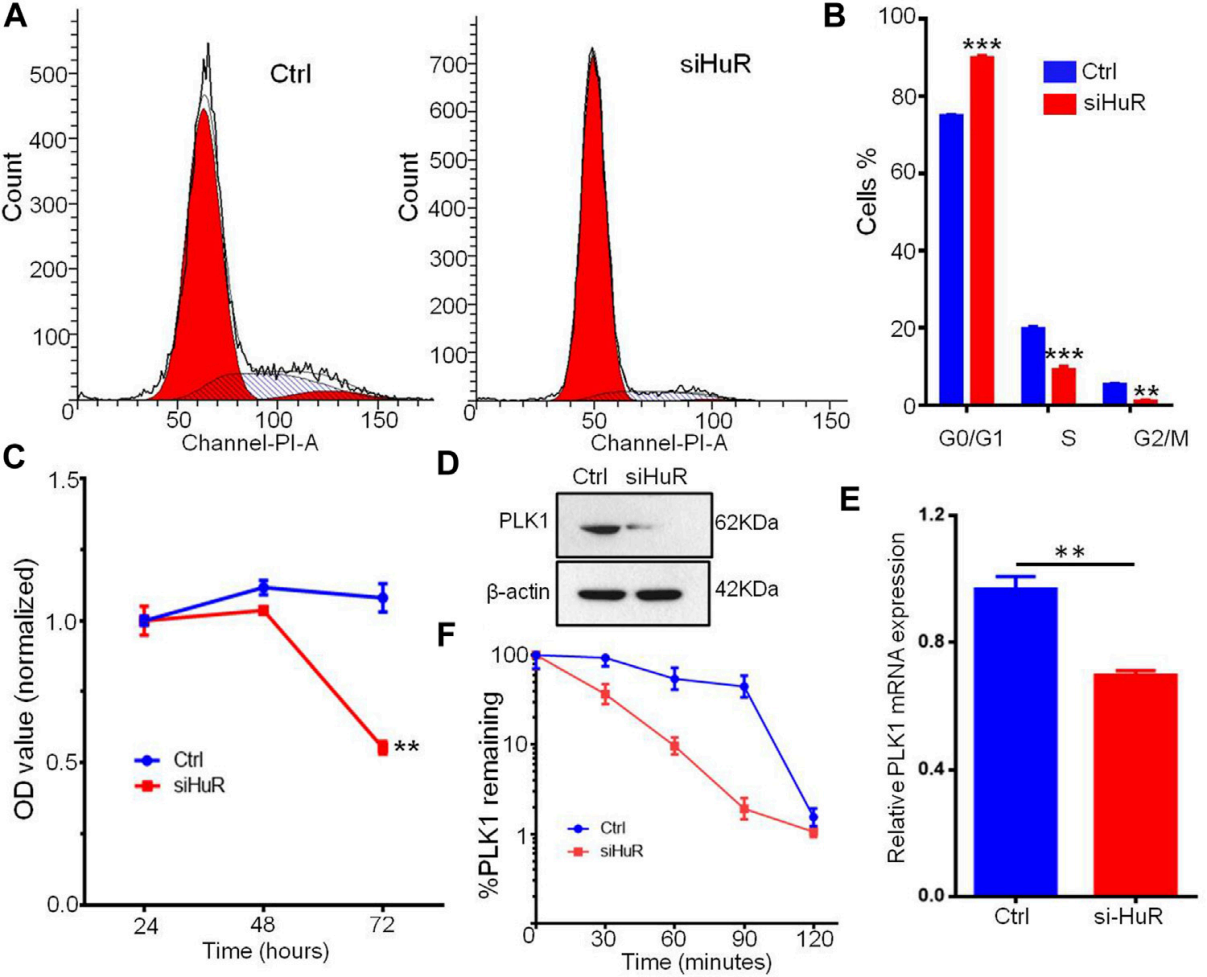
HuR promotes the cell cycle progression and proliferation of A549 cells. **(A,B)** Cell cycle histograms of A549 HuR KD cells compared with A549 cells. **(C)**. CCK-8 assay of the proliferation of A549 cells and HuR KD A549 cells. **(D)**. PLK1 was measured by western blotting, and to normalize protein loading, the blots were also probed for β-actin. **(E)**. Relative PLK1 mRNA expression in A549 cells with HuR KD for 24 h **(F)**. The decay rates of the PLK1 and GAPDH mRNA were assessed in A549 cells by RT-qPCR at different time points (30, 60, 90, 120 min) after transcription inhibition with actinomycin D. Data are represented as the mean ± SEM. *p < 0.05, **p < 0.01, ***p < 0.001. *n* = 3.

To find the most effective siRNA for knocking down HuR expression, we chose three different siRNA sequences and detected their efficiencies by real-time PCR (Figure 5A) and western blotting (Figure 5B). We then explored the role of HuR and found that it can promote the migration and invasion of A549 cells compared with that of A549 HuR KD cells (Figures 5C,D). Statistical results are shown in Figures 5E,F. Next, we evaluated the effect of HuR on the proliferation of A549 cells (Figure 6C).

### HuR Regulates PLK1 mRNA Levels and Protein Expression

PLK1, an essential cell cycle regulator, plays a key role in regulating the cell cycle by compromising cell cycle checkpoints and increasing the expression of chromosomal instability (CIN)-related genes. We assumed that the changes in PLK1 expression might be a possible mechanism by which G1/S and G2/M transition occurs during cell growth induced by HuR. Flow cytometry analysis showed that HuR increased the number of cells in the S phase of the cell cycle compared to that in the untreated group (Figures 6A,B). Next, we examined whether the decrease in PLK1 protein expression mediated by HuR was correlated with a decrease in the PLK1 transcript level. To this end, quantitative RT-PCR of PLK1 was performed on control or HuR KD A549 cells 2 days after transfection. HuR KD decreased the expression of PLK1, as indicated by western blot analysis (Figure 6D). In treated cells, we showed that HuR treatment led to a decrease in PLK1 mRNA levels by 2-fold (Figure 6E).

Next, to examine the PLK1 mRNA half-life (*t*_1/2_) in A549 cells treated with HuR KD, actinomycin D inhibitor was applied, and the level of PLK1 mRNA was measured by RT-qPCR at 0, 30, 60, 90 and 120 min. In HuR KD A549 cells, the PLK1 mRNA *t*_1/2_ was 33 min, whereas, in control A549 cells, PLK1 mRNA *t*_1/2_ was 97 min. The mRNA *t*_1/2_ of GAPDH (the control) was not changed.

## Discussion

Lung cancer is a leading cause of cancer-associated mortality worldwide, with a 5-year survival rate of <15% (Siegel et al., 2021). Proliferation is the leading factor influencing cancer development (Liu et al., 2021b; Hou et al., 2021; Wang et al., 2021). HuR is the key molecule in lung cancer progression (Liao et al., 2011; Li et al., 2013; Zhang et al., 2020). However, little is known about how HuR might act to specifically promote lung cancer proliferation. In this study, the expression of HuR was significantly associated with lung cancer aggressiveness; for example, lung patients with high expression of HuR showed a poor prognosis in the TCGA database. Here, we also found that the main function of HuR in the lung adenocarcinoma cell line A549 was related to ribonucleoprotein complex biogenesis. The signaling pathways that regulated HuR in A549 cells were mainly related to the spliceosome, RNA transport, and the cell cycle. Furthermore, our results suggest that HuR can suppress the protein and mRNA expression of PLK1 in A549 cells, which may promote cell cycle progression and cell proliferation, migration, and invasion. Overall, these data suggest that HuR is overexpressed in lung cancer patients and related to poor prognosis. HuR plays an important role in lung cancer progression as well.

In this study, we confirmed that HuR was overexpressed in patients with lung cancer versus those with benign lung diseases, such as benign pulmonary nodules and tuberculosis. In addition, the IRS of HuR in lung cancer tissue was found to differ markedly from that in tissue from patients with benign lung diseases. These findings emphasize that lung cancer is a complex disease; in particular, lung proliferation is a very important step in the progression of cancer. Kaplan-Meier analysis of overall survival according to HuR expression was performed by employing the TCGA. Lung cancer patients with high expression of HuR had a poor prognosis. This suggests that the overexpression of HuR in lung cancer increases the risk of metastasis. Wang *et al* proposed that UDP-glucose regulates lung cancer metastasis and uncovered a mechanism by which UDP-glucose 6-dehydrogenase promotes tumor metastasis by increasing the association of HuR with SNAI1 mRNA and therefore stabilizing SNAI1 mRNA (Wang et al., 2019).

Our results show for the first time the function of HuR in lung adenocarcinoma cells, and HuR KD A549 cells were analyzed by single-cell RNA sequencing analysis. HuR KD in A549 cells yielded 2,466 upregulated genes and 3,114 downregulated genes. The main functions of HuR in A549 cells were related to ribonucleoprotein complex biogenesis. The signaling pathways were mainly related to the spliceosome, RNA transport, and the cell cycle.

Little is known about the functionality of HuR in lung cancer. In the current study, we assessed the proliferation, migration, and invasion of A549 cells after HuR KD. We confirmed that HuR can enhance the proliferation of A549 cells and promote their migration and invasion. The KD of HuR did not dramatically enhance proliferation, migration, or invasion. These results indicate the enhancing effect of HuR on lung cancer cell proliferative abilities. This finding is consistent with that of Muralidharan R et al, who found that targeted inhibition of HuR in cancer cells suppresses several HuR-regulated oncoproteins, increasing anticancer treatment efficacy (Muralidharan et al., 2016).

We detected the cell cycle distribution in A549 cells and explored potential related mechanisms. A549 control cells showed accelerated S phase transition from the G1 phase compared with HuR KD cells, which were arrested in the G1 phase. PLK1 is a well-known regulator of the cell cycle and can arrest cells in the G1/S phase by blocking the G2/M transition. In this study, we showed that KD of HuR led to the downregulation of PLK1. The HuR can stable PLK1 mRNA was shown by western blotting, RT-PCR, and mRNA decay assays. However, a study by de Carcer G et al. showed that PLK1 overexpression can prevent the development of Kras-induced and Her2-induced mammary gland tumors, which exhibit high levels of CIN (de Cárcer et al., 2018). Overall, our data imply that HuR can influence the cell cycle signaling pathway in lung cancer cells. This further corroborated the findings of our study. However, this hypothesis regarding the downstream function of PLK1 is beyond the scope of this study.

This study does have some shortcomings. In the clinical specimen analysis, we did not conduct further analysis of clinical data for patients with high HuR expression. *In vitro* experiments, the detailed mechanism of the downregulation of PLK1 and inhibition of cell cycle progression after HuR KD was not further explored.

In summary, our data showed that HuR overexpression in lung cancer was related to a poor prognosis. Furthermore, our study showed that HuR can enhance cell proliferation, migration, and invasion, which could enhance the expression of PLK1 in A549 cells. Based on our results, further studies are necessary to explore how HuR effect on the downstream molecular in lung cancer.

## Data Availability

The datasets presented in this study can be found in online repositories. The names of the repository/repositories and accession number(s) can be found below: https://www.ncbi.nlm.nih.gov; PRJNA767760.

## Abbreviations

FBS, fetal bovine serum; HuR, human antigen R; IRS, immunoreactivity score; 3′-UTR 3′, untranslated region; KD, knockdown.
